# Biofilm and Spore Formation of *Clostridium perfringens* and Its Resistance to Disinfectant and Oxidative Stress

**DOI:** 10.3390/antibiotics10040396

**Published:** 2021-04-06

**Authors:** Wen Si Hu, Dong U Woo, Yang Jae Kang, Ok Kyung Koo

**Affiliations:** 1 Department of Food Science and Engineering, Liaocheng University, Liaocheng 252059, China; huwensi@naver.com; 2 Division of Life Science Department, Gyeongsang National University, Jinju 52828, Korea; dongu7610@naver.com (D.U.W.); kangyangjae@plantprofile.net (Y.J.K.); 3 Division of Bio & Medical Big Data Department (BK4 Program), Gyeongsang National University, Jinju 52828, Korea; 4 Department of Food and Nutrition, Gyeongsang National University, Jinju 52828, Korea; 5 Institute of Agriculture and Life Science, Gyeongsang National University, Jinju 52828, Korea

**Keywords:** *Clostridium perfringens*, biofilm, spore, disinfection, oxidative stress

## Abstract

*Clostridium perfringens* is a major human pathogen that causes gastroenteritis via enterotoxin production and has the ability to form spores and biofilms for environmental persistence and disease transmission. This study aimed to compare the disinfectant and environmental resistance properties of *C. perfringens* vegetative cells and spores in planktonic and sessile conditions, and to examine the nucleotide polymorphisms and transcription under sessile conditions in *C. perfringens* strains isolated from meat. The sporulation rate of sessile *C. perfringens* TYJAM-D-66 (*cpe*+) was approximately 19% at day 5, while those of CMM-C-80 (*cpe*−) and SDE-B-202 (*cpe*+) were only 0.26% and 0.67%, respectively, at day 7. When exposed to aerobic conditions for 36 h, TYJAM-D-66, CMM-C-80, and SDE-B-202 vegetative cells showed 1.70 log, 5.36 log, and 5.67 log reductions, respectively. After treatment with sodium hypochlorite, the survival rates of TYJAM-D-66 vegetative cells (53.6%) and spores (82.3%) in biofilms were higher than those of planktonic cells (9.23%). Biofilm- and spore-related genes showed different expression within TYJAM-D-66 (–4.66~113.5), CMM-C-80 (–3.02~2.49), and SDE-B-202 (–5.07~2.73). Our results indicate the resistance of sessile cells and spores of *C. perfringens* upon exposure to stress conditions after biofilm formation.

## 1. Introduction

*Clostridium perfringens* is a Gram-positive, anaerobic, spore-forming pathogen that causes intestinal diseases in humans and animals [1,2]. Its virulence is primarily derived from the ability of this organism to produce at least 20 different toxins, potentially causing gas gangrene in contaminated wounds, gastroenteritis in humans, and necrotic enteritis in chickens. The classification of *C. perfringens* depends on the production of alpha (*cpa*), beta (*cpb*), epsilon (*etx*), iota (*iap*), enterotoxin (*cpe*), and necrotic beta-like (*netB*) genes [3]. Historically, *C. perfringens* strains were classified based on five toxin types (types A to E), but the classification was recently expanded to seven toxin types (types A to G) [4]. *C. perfringens* type F is a global food safety concern, which is responsible for the second most commonly reported type of foodborne illness in the USA, Canada, and some developing countries [4,5].

Bacterial biofilms is a food safety concern because of its persistence and resistance to the environment. Biofilms are defined as aggregates of planktonic cells that are embedded in extracellular polymeric substances (EPSs) that protect microorganisms from environmental stress [6]. Multiple regulatory processes contribute to controlling gene expression during biofilm formation by *C. perfringens*. Approximately 25.7% of the genes in *C. perfringens* exhibit differential expression between biofilms and planktonic cells, including genes associated with EPS biosynthesis (*epsA-O, tasA, tapA, bslA, sipW,* and *calY*) [7], toxin production (*virX, luxS, codY,* and *ctrAB*) [8,9], motility (*ccpA*) [10], sporulation (*sigG, sleC, soj, spo0A, spoVB, spoVD*, and *spoIIE*) [11,12], and the stress response (*hrcA, lexA*, and *recA*) [13]. These genes are involved in cell metabolism, transcription, translation, and chromosomal replication, and are downregulated in biofilms. Biofilms can lead to decreased cell growth and division by allowing cells to conserve energy, prolonging the lifespan of cells [14]. Virulence genes encoding alpha-clostripain, collagenase, necrotic enteritis toxin B, perfringolysin O, and phospholipase C are downregulated, suggesting that the biofilm results in a quiescent growth mode and that these toxin proteins are not necessarily involved in biofilm formation [13]. However, hemolysins (such as alpha toxin and perfringolysin O) were upregulated in the biofilm and are detected in a complex with eDNA, showing a potential increase in its pathogenicity [15].

The majority of cells in biofilms are in a vegetative state, but sporulation might occur during biofilm formation and persistence [16]. Sporulation is essential for the production of *C. perfringens* enterotoxins (CPEs). Specifically, the production of CPE is controlled by the master sporulation regulator Spo0A, the transcriptional regulator NanR, and three sigma factors, SigE, SigF, and SigK [9,17]. The transcriptional regulator of epsilon-toxin, *codY*, was also previously linked to sporulation [8]. Spo0A regulates both sporulation and biofilm formation via phosphorylation and interaction with orphan histidine kinases [16,18].

Previous studies attempted to develop effective inactivation strategies against vegetative *C. perfringens*, using physical and chemical preservatives, and naturally derived antimicrobial agents [6]. However, there are few studies on the resistance of *C. perfringens* vegetative cells and spores, as disinfectants after biofilm formation. The objective of this study was to compare the spore forming ability of different isolates over time during biofilm formation, and their resistance to disinfectant and oxidative stress after spore or biofilm formation. In order to find the correlation between spore and biofilm formation, and their resistance to the stress conditions among isolates, we compared the single nucleotide polymorphisms (SNPs), and the transcriptional profiles during biofilm formation.

## 2. Results

### 2.1. Biofilm Formation, Sporulation, and Toxin Gene Profiles of C. perfringens Strains

All *C. perfringens* isolates showed strong biofilm formation abilities measured with a crystal violet assay from a previous study, with the absorbance of 2.94, 1.96, and 1.17 on TYJAM-D-66, CMM-C-80, and SDE-B-202, respectively [6]. TYJAM-D-66, carrying the *C. perfringens* enterotoxin gene, exhibited a spore formation rate of 7.36%. *C. perfringens* ATCC 13124 exhibited a spore formation rate of 0.13%, and CMM-C-80 and SDE-B-202 had no sporulation activity after 24 h. These isolates were selected for further investigation of biofilms and sporulation—CMM-C-80, which exhibits a different genetic profile from TYJAM-D-66, which carries *netB*, but not the *cpe* gene, and SDE-B-202, which exhibits a similar toxin profile (*cpe*+) as TYJAM-D-66 [6].

### 2.2. Sporulation Efficiency during Biofilm Formation

**Figure 1:**
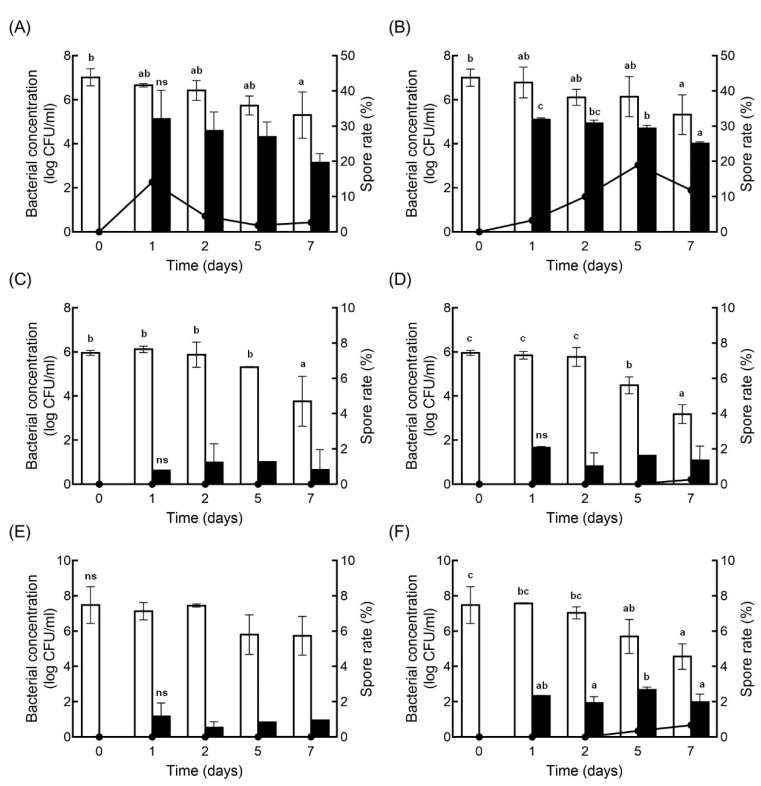
Vegetative cell counts and sporulation dynamics in biofilms. Vegetative cell (□) and spore (▪) counts of *C. perfringens* TYJAM-D-66 (**A**,**B**), CMM-C-80 (**C**,**D**), and SDE-B-202 (**E**,**F**) under planktonic (**A**,**C**,**E**) and sessile (**B**,**D**,**F**) conditions, over 7 days of biofilm formation. Spore rates were determined as the average ratio of the spores to sessile cells, and are represented by a line (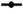). The presented values are the averages of at least three replicates. Different lowercase alphabets indicate significant differences between groups at the time-points (*p* < 0.05).

**Figure 2:**
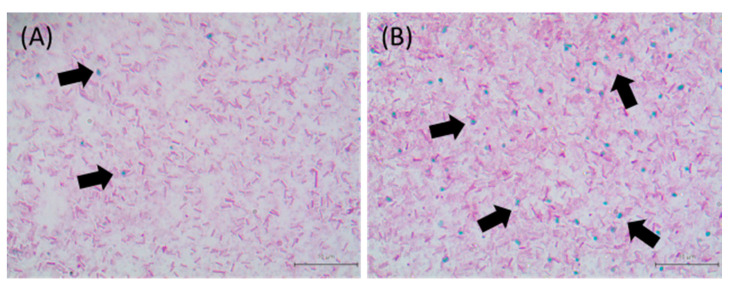
Microscopy images of TYJAM-D-66 sporulation in planktonic (**A**) and sessile (**B**) cells incubated for 3 days in a DS medium. Spores stained with malachite green appear green/blue (black arrows), and vegetative cells colored with safranine in red. Images were obtained using an optical microscope at a magnification of 1000*×*.

The sporulation efficiency was higher under sessile conditions than under planktonic conditions at day 5 and day 7, in all isolates (Figure 1). CMM-C-80 and SDE-B-202 showed very low sporulation efficacies of 0.26 and 0.67%, respectively, at day 7. The image in Figure 2 shows the presence of spores in sessile and planktonic conditions.

### 2.3. Resistance of C. perfringens Vegetative Cells and Spores to Oxidative Stress

**Figure 3:**
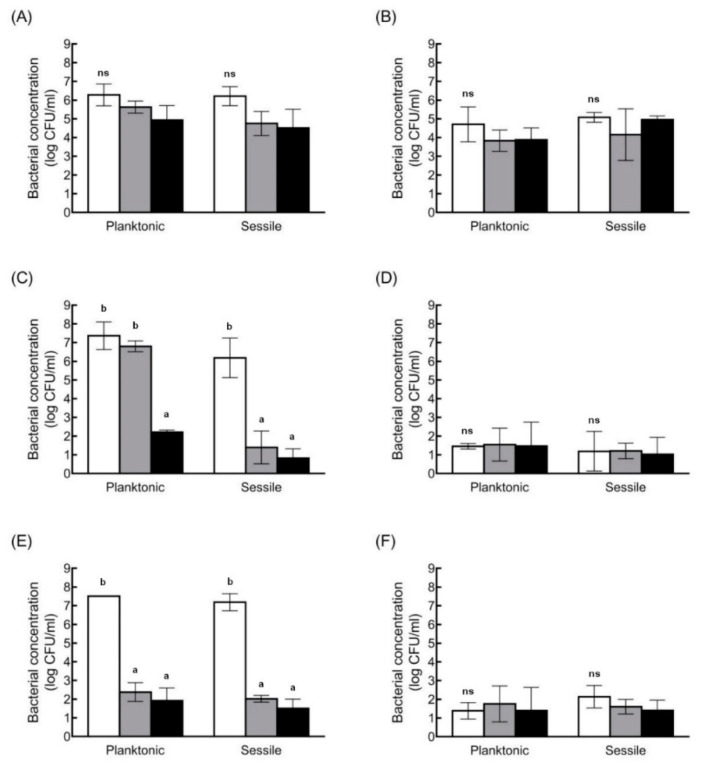
Survival of *C. perfringens* vegetative cells and spore resistance under oxidative stress. Bacterial concentrations of TYJAM-D-66 (**A**,**B**), CMM-C-80 (**C**,**D**), and SDE-B-202 (**E**,**F**) in vegetative cells (**A**,**C**,**E**) and spores (**B**,**D**,**F**), after exposure to atmospheric oxygen for 0 h (□), 18 h (▪), and 36 h (▪). Different lowercase alphabets indicate significant differences between groups at same conditions (*p* < 0.05).

*C. perfringens* isolates were exposed to atmospheric conditions to evaluate the resistance of these isolates to oxidative stress (Figure 3). After 36 h, the planktonic cells of TYJAM-D-66, CMM-C-80, and SDE-B-202 showed 1.35 log, 5.13 log, and 5.59 log reductions in vegetative cells, and the sessile cells showed 1.70 log, 5.36 log, and 5.67 log reductions, respectively. There was no significant difference in the surviving bacterial concentrations between planktonic and sessile cells after 36 h. The spores of TYJAM-D-66 were 4.71 log CFU/mL and 5.08 log CFU/mL in planktonic and sessile conditions, respectively, and there were under 1 log reduction after 36 h. CMM-C-80 and SDE-B-202 spore counts showed 1.46 log CFU/mL and 1.39 log CFU/mL in planktonic, 1.19 log CFU/mL, and 2.14 log CFU/mL in sessile conditions, respectively, with no significant change under the oxidative stress. In addition, planktonic and sessile conditions had little or no effect on sporulation during 36 h of oxygen exposure. TYJAM-D-66 was significantly more tolerant to atmospheric condition than CMM-C-80 and SDE-B-202. The survival rates of planktonic CMM-C-80 and SDE-B-202 decreased significantly after 36 h and 18 h of exposure to oxygen, respectively. These results demonstrated that strain TYJAM-D-66 was able to survive under oxygen exposure. Although the biofilm cell numbers were very similar in all cultures, oxygen tolerance differed among the isolates.

### 2.4. Resistance of C. perfringens Vegetative Cells and Spores to Disinfectants

**Figure 4:**
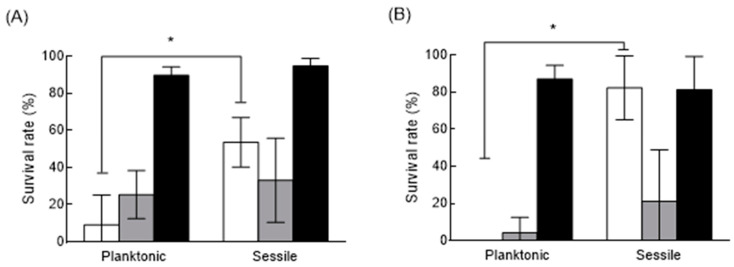
Survival of *C. perfringens* vegetative cells and spores among planktonic and sessile cells after exposure to disinfectants. Survival rate of TYJAM-D-66 vegetative cells (**A**) and spores (**B**) grown after exposure to 0.001% sodium hypochlorite (□), 5% hydrogen peroxide (▪), and 80% ethyl alcohol (▪) for 10 min. The asterisk represents statistical significance (*p* < 0.05) among experimental groups.

The survival of *C. perfringens* TYJAM-D-66 in the presence of different disinfectants was evaluated (Figure 4). TYJAM-D-66 was selected based on the higher spore rate in biofilms and its resistance to the oxidative stress, as compared to other isolates. Due to the differences in the initial cell and spore concentrations, the survival rate was used to evaluate disinfectant susceptibility. The survival rates of the planktonic TYJAM-D-66 cells were 9.23%, 25.3%, and 89.7% after treatment with sodium hypochlorite, hydrogen peroxide, and ethyl alcohol, respectively, whereas the survival rates of the vegetative cells in biofilms were 53.6%, 33.1%, and 98.2%, respectively. TYJAM-D-66 sessile cells exhibited significantly higher survival rates than planktonic cells when treated with sodium hypochlorite and hydrogen peroxide, while ethyl alcohol showed poor disinfection with no statistically significant difference between planktonic and sessile cells. The spores exhibited higher survival rates under sessile conditions, upon treatment with sodium hypochlorite. 

### 2.5. SNP Discovery Analysis

**Figure 5:**
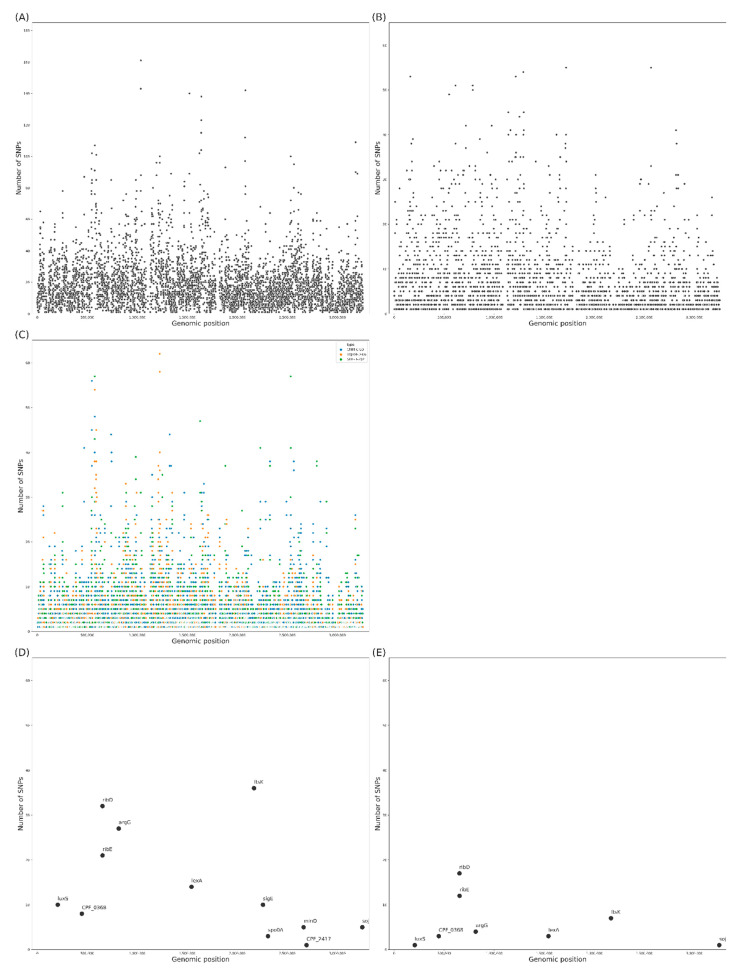
SNP count characterization summary. Total SNP counts within CDSs (**A**) and intergenic regions (**B**). The nonsynonymous SNP counts of *C. perfringens* TYJAM-D-66 (blue), CMM-C-80 (orange), and SDE-B-202 (green) in CDSs (**C**). Total (**D**) and nonsynonymous (**E**) SNP counts in the selected genes within CDSs.

A summary of the obtained SNP results for each of the classifications according to the genomic regions can be found in Figure 5. A total of 124,556 SNPs were identified (in all gene regions) from three isolated *C. perfringens* strains. Among these SNPs, 102,466 SNPs (Figure 5A) were found within coding sequences (CDS), and the remainder (22,090 SNPs) were found to be intergenic SNP (Figure 5B). The numbers of nonsynonymous SNPs found in CDSs of TYJAM-D-66, CMM-C-80, and SDE-B-202 were 13,502, 18,141, and 16,944, respectively (Figure 5C). To investigate the genetic diversity of sporulation and biofilm formation, a total of 15 genes were selected, as shown in Figure 5D,E. Based on the correlated functional gene states in the three strains, 194 SNPs (Figure 5D) and 51 nonsynonymous substitutions (Figure 5E) were identified. The results provided concrete data for each related gene site, and the number of nonsynonymous SNPs in *ribD, ribE,* and *ftsK* were 17, 12, and 7, respectively. In the analyses of the putative amino acid sequences, the homology with the biofilm formation related proteins were ArgG (99.8%), CPF_0368 (99.4%), RibD (98.5%), RibE (98.5%), and LexA (99.1%), and with sporulation related proteins, these were FtsK (99.8%) and Soj (99.9%) in three isolates and ATCC 13124 (data not shown).

### 2.6. Expression Changes in C. perfringens Planktonic and Sessile Cells

The differential transcriptional levels of biofilm formation and sporulation related genes were tested through RT–PCR. The genes were selected from previous studies on *C. perfringens* sporulation and biofilm formation [11,13]. Among 11 biofilm-related genes, 10 genes showed increased expression in TYJAM-D-66 (Table 1). The *sigG, argG*, and *ribD* gene expressions in TYJAM-D-66 were increased significantly with fold changes of 45.0, 113.5, and 42.8, respectively. CMM-C-80 and SDE-B-202 showed significantly lower expression or downregulations, with fold changes ranging from –5.64 to 6.60. All 10 sporulation-related genes were upregulated in TYJAM-D-66. The *sigE, sigK, spollAA,* and *spollE* in TYJAM-D-66 showed significant increases in expression of 23.2, 70.2, 22.1, and 58.2 fold, respectively. In CMM-C-80, 2 genes were upregulated, and the fold changes of sporulation-related genes ranged from –2.40 to 2.49. SDE-B-202 showed downregulation of all tested genes, with fold changes of –5.07 to –1.95.

## 3. Discussion

Microorganism contamination can occur through many routes in food processing facilities, where pathogens can survive and persist in the environment and in equipment. Biofilms and spores restrict antimicrobial penetration and contribute to the recalcitrance of bacterial infections [19,20]. There are several hypotheses regarding whether the pathogenicity of foodborne pathogens is affected when they survive in the environment via biofilm and spore formation. In gastrointestinal infections, CPEs are produced and accumulate during spore formation and are released from mother cells [9]. However, CPE synthesis is probably not required for normal spore production, since many CPE-negative *C. perfringens* strains are also capable of sporulation [2,21]. In our study, all three tested strains of TYJAM-D-66 (*cpe*+), CMM-C-80 (*cpe*−), and SDE-B-202 (*cpe*+) exhibited strong biofilm formation and the ability to produce spores. These results suggest that the presence or absence of the *cpe* did not determine the capacity for the sporulation of biofilm formation. Castro et al. (2016) reported the absence of CPE expression in planktonic and sessile cells, suggesting that CPE might not be essential for biofilm formation [22]. Therefore, while CPE is an important virulence factor for *C. perfringens*, it was not considered in this study.

Cells can induce sporulation upon the sensing of external stimuli, nutrient deficiencies, and high cell densities, and the sporulation rate might differ in each strain [23,24]. Previous studies showed a wide range of biofilm formation or sporulation efficiencies for *C. perfringens* isolates, with an absorbance range between 0 and 0.9 for biofilm, and values greater than 80% or less than 10% of the sporulation rates [5,9]. Spores bind to a surface and agglomerate over time with increasing hydrophobicity, which further supports the higher sporulation rate in biofilms than in planktonic conditions [25]. Other studies suggested that the spore germination rate is lower in biofilms than in planktonic cultures of *C. difficile* [26]. Our study indicated that the sporulation rate in biofilms continued to increase and then stabilized with a higher sporulation rate than in planktonic conditions. In addition, limited nutrients caused the gradual bacterial death, especially on planktonic cells during incubation. This might be the reason that the increase of sporulation rate did not increase further, after 24 h of incubation.

*C. perfringens* is an anaerobic bacterium, however, it can survive under occasional oxidative stress for short durations. Oxygen is toxic to strictly anaerobic vegetative cells, due to the formation of superoxide radicals, hydrogen peroxide, or hydroxyl radicals [27]. However, the death of *C. perfringens* under oxidative stress is not rapid. *C. perfringens* possesses specialized genes such as *sod* and *ahpC*, with a hypothetical superoxide reductase activity [28]. Increased expression of oxidative stress resistance genes (CPF_0904, and *vhb*) encoding gluthatione peroxidase, and hemoglobin was observed in *C. perfringens* biofilms [13]. Some of these genes (*ydaD*, *ycdF*, *uidA*, *ydaB*, and *ydb*) were involved in the oxidative stress in *C. perfringens* [27]. In our results, TYJAM-D-66 showed the highest resistance to oxidative stress, however, the above-mentioned genes did not contain any SNPs in the tested strains. Other genes might be involved in the response of oxidative stress or might indirectly regulate gene expression. The *ribD* showed high expression in TYJAM-D-66, which is linked to the secretion of flavin mononucleotide (FMN). The FMN is a well-known free flavin, which belongs to the EPSs ingredient and acts as electron shuttles involved in the oxidative stress response and enhanced biofilm formation [29]. Some vegetative cells are protected by biofilms and stress-induced protective genes, contributing to their survival. In the atmospheric oxygen tolerance assays, the *C. perfringens* planktonic cells exhibited 63 and 7.4% viability after 6 and 24 h of exposure to oxygen, respectively [30]. We inferred that the resistance to oxidative stress was proportional to biofilm formation and the biofilms could protect *C. perfringens* from oxidative stress. This finding might explain the high resistance of TYJAM-D-66 to oxidative stress. However, the results obtained from SDE-B-202 showed that the two parameters were not correlated and it is more affected in a strain-dependent manner.

Disinfection with chlorine and oxygen containing disinfectants is commonly used in the food industry, with varying degrees of success for killing vegetative cells and spores [31]. In the presence of 10% hydrogen peroxide, the viability of a biofilm was more than 35% higher than that of planktonic cells of *C. perfringens*, and the viability of *C. perfringens* treated with sodium hypochlorite was 14.1% for planktonic cells and 3.8% for biofilms [5]. Organic substances react with the available chlorine in the biofilm, decreasing the availability of chlorine for disinfection [32]. In our study, *C. perfringens* showed a significantly higher survival rate against sodium hypochlorite under sessile conditions, and it was even higher with the spores in biofilm. Sodium hypochlorite is the most commonly used sanitizer in foodservice facilities, however, this study showed a significant defect when used on biofilm and spore-formed pathogen. Hydrogen peroxide was more efficient in reducing *C. perfringens* vegetative cells and spores counts in sessile conditions, as compared to sodium hypochlorite. Sodium hypochlorite and hydrogen peroxide could liberate active chlorine and hydroxyl free radicals, respectively, which are powerful oxidizing agent that inactivates the bacterial enzymes to kill bacteria in a very short time [5,33]. The factors involved in *C. perfringens* spore resistance include the characteristics, like spore core water content, cortex peptidoglycan structure, pyridine-2,6-dicarboxylic acid level, and saturation of α/β-type small, acid-soluble spore proteins on DNA [34,35]. However, the mechanism of the killing of *C. perfringens* spores by hydrogen peroxide is still obscure. Paredes-Sabja et al. found that *spoVA* mutations increased *C. perfringens* spore resistance to hydrogen peroxide significantly [35]. Ethanol can denature and coagulate protein, but the coagulated protein might also prevent the ethanol from penetrating deeper into the bacteria, which could affect the germicidal efficacy [36]. *C. perfringens* spore is observed to show sensitivity to nitrous acid, formaldehyde, HCl, and UV radiation [34,35].

Recently, researchers indicated that there is a strong interconnection between sporulation and biofilm formation [12,37]. A complex regulatory circuit is implicated in the expression of many different genes and master regulators, such as *spo0A, lexA,* and *sinR*, in which they are associated with biofilm formation and sporulation [17,38,39]. The observation of biofilm formation in *Bacillus cereus* at different cell stages showed that spores hold great potentials for initial attachment for biofilm formation [40]. SDE-B-202 showed a strong biofilm formation ability, in which *argG* was the only upregulated gene among all tested genes, which was evident in the other three strains. *argG* produces argininosuccinate synthase, which are associated with amino acid metabolism and transport, and has a positive impact on biofilm formation. The results demonstrated that *argG* was not overexpressed in all biofilms formed by *C. perfringens*. The RT–PCR level of individual genes represented the relative gene expression, and the correlation to the degree of biofilm formation, but this was not absolute.

TYJAM-D-66 exhibited a high rate of sporulation, and the spore-related genes showed increased expression of *sigK*, *spollE*, *sigE*, *spoVD*, and *spollAA.* The SpoIIIE and ftsK are DNA translocase, which play an important role in cell division and sporulation by transferring DNA from the cell to the forespore [41]. Sigma factors *sigK* mutant strains were determined to regulate the enterotoxin synthesis and sporulation process of *C. perfringens* [42]. In this study, the nonsynonymous SNPs of *lexA* and *ribE* gene were distinctively higher in TYJAM-D-66, as compared to the other strains (Supplementary Table S1). The *C. difficile luxS* mutant was unable to form biofilm on glass surface [16]. luxS is a major modulator of quorum sensing, which is responsible for autoinducer 2 production [43]. The ribE gene encodes the alpha subunit of riboflavin synthase, and its derivatives were proven to promote biofilm formation in *Salmonella enterica* [44]. Whole-genome sequence (WGS) analysis is suitable for identifying and analyzing the biochemical characteristics of pathogen strains, which is beneficial for understanding the genetic aspects of antimicrobial resistance, transmission patterns, and virulence [45,46]. WGS was used to characterize relapse with increased certainty of *C. difficile* infection and distinguish relapse from reinfection in clinical patients, even if one single nucleotide variants also impact gene function [47]. Interestingly, a WGS study with the *C. perfringens* virulence factors on necrotic enteritis showed different infections of turkeys and chickens [48]. The SNPs were distributed across several different gene categories, according to genome sequence comparison. The SNP is a variation in genetic sequence that leads to amino acid sequences and structure changes. The nonsynonymous SNPs are particularly important, as they represent the differences in the translated amino acid sequence. The mutation appearance in regulators of virulence can dramatically alter bacterial physiology, causing adverse clinical outcomes [49]. We particularly focused on biofilm- and spore-related genes to examine whether the differences in gene frequencies indicate the different physiological characteristics between *C. perfringens* isolates. This research suggests that further phylogenetic studies involving WGS and the surveillance of various derived *C. perfringens* isolates should be conducted, which would require a deep exploration of their biological characteristics, so that we can establish successful interventions or preventive measures.

## 4. Materials and Methods

### 4.1. Bacterial Strains and Growth Conditions

*C. perfringens* stains TYJAM-D-66, CMM-C-80, and SDE-B-202 (Table 2) used in this study were isolated from meat collected from a school cafeteria [6]. The *C. perfringens* strains were cultured in TGY broth containing 3% trypticase (Millipore, Billerica, MA, USA), 2% glucose (Junsei Chemical Co., Ltd., Tokyo, Japan), 1% yeast extract (Becton Dickinson and Company, BD, Sparks, MD, USA), and 0.1% L-cysteine (Junsei Chemical Co.), overnight at 37 °C under anaerobic conditions.

### 4.2. Spore Quantification

The overnight cultures (2%) were transferred to a 5 mL Duncan-Strong (DS) sporulation medium (HiMedia Laboratories Pvt Ltd., Mumbai, India) broth in 14 mL round bottom tubes (SPL Life Science Co. Ltd., Pocheon, Korea), followed by incubation for 24 h at 37 °C, under anaerobic conditions. Sporulation under planktonic and sessile conditions was determined after 0, 1, 2, 5, and 7 days at 37 °C. The sporulation at day 0 was served as a negative control. The vegetative cells were plated on brain heart infusion (BHI) agar (BD) for a total count quantification. For spore quantification, the culture was heated at 80 °C for 10 min, cooled in water at room temperature for 30 min, and subsequently incubated at 40 °C for 30 min, for spore germination. The treated cells were plated on BHI agar, and the heat-resistant spores were counted. The experiments were performed in duplicates, with three separate experiments. The spore rate was expressed as the ratio of the spore counts to the vegetative cell counts, as given in the following formula.

(1) $$\mathrm{Spore}\;\mathrm{rate}\;\left(\%\right)=\frac{\mathrm{Spore}\;\mathrm{counts}}{\begin{array}{c} \mathrm{Vegetative}\;\mathrm{cell}\;\mathrm{counts} \end{array}}\times 100$$

### 4.3. Microscopy Analysis of Spores

The spores were observed using an optical microscope. The planktonic and sessile cells were collected by centrifugation and fixed with heat on glass slides. The cells were stained with 5% malachite green (Daejung Chemicals and Metals Co. Ltd., Siheung, Korea), placed over a beaker on which they were heated by steam from boiling water for 30 min, after which they were rinsed with softened water. Vegetative cells were then counterstained with 2% safranine (BD) for 10 min at room temperature, and the remaining cells were rinsed with softened water. The stained cells were viewed under an optical microscope (Model Eclipse Ci-L, Nikon Corporation, Tokyo, Japan) at 1000*×* magnification, and analyzed using the i-Solution Lite software program (IMT i-Solution, Inc., Vancouver, BC, Canada). The dyeing experiment was performed at least in duplicates, with two independent experiments.

### 4.4. Oxidative Stress and Disinfectant Treatments

*C. perfringens* strains were incubated in six-well polystyrene plates at 37 °C for 48 h, under anaerobic conditions. After incubation, sessile cells were collected by removing the medium and washed with PBS one time, and the planktonic cells were obtained by centrifugation. The initial cell concentration of vegetative cells and spores were 10^5^ CFU/mL and 10^4^ CFU/mL, respectively. The cells were exposed to disinfectants for 10 min or to atmospheric conditions for 18 or 36 h. In the negative control, the disinfectants were not treated. Three types of disinfectants that are commonly used in the food processing industry were tested on TYJAM-D-66 in this study, at concentrations suggested by the manufacturers—0.01% sodium hypochlorite (Junsei Chemicals Co., Chuo-ku, Tokyo), 5% hydrogen peroxide (H_2_O_2_) (Daejung), and 80% ethyl alcohol (Duksan Pure Chemicals Co., Ltd., Ansan, Korea). After treatment, the vegetative cells and the germinated spores were determined by plating onto the BHI agar medium. The plates were incubated anaerobically at 37 °C for 24 h. After incubation, the colonies were counted to determine the surviving population size. The experiments for each strain were performed three times with duplicates.

### 4.5. Whole-Genome Sequencing and Variant Calling Analysis

Multiplex libraries were generated with TruSeq sample preparation kits (Illumina, San Diego, CA, USA) and were sequenced with Illumina MiSeq sequencer chemistry, generating paired-end reads of 300 base pairs (bp) each. All sequencing data generated for this project are available through bioproject accessions PRJNA634218 at the National Center for Biotechnology Information (NCBI, Bethesda, MD, USA). The reads were trimmed, and low-quality bases were filtered with Trimmomatic (version 0.39), followed by alignment to the reference sequence of *C. perfringens* strain ATCC 13124 (NCBI Accession No. NC_008261.1) using the Burrows-Wheeler Aligner (BWA version 0.7.12) program. The open reading frames (ORFs) region was confirmed in the reference genome’s Genomic GFF information [50]. The genomic sequences were divided into subsequences of 1000 bp, with the overlapping fragment size set at 500 bp. We identified SNPs by extracting single-base pair mismatches from the alignment of the two genomes.

### 4.6. Nonsynonymous Substitution

To further explore the genomic similarities among isolates, we clustered 3 isolates and additional *C. perfringens* strains (the sequencing reads were downloaded from NCBI under run accessions of SRP128122 and SRP155315), for nonsynonymous substitution of SNPs, and the SNPs included in the candidate genes. The functional characteristics of *C. perfringens* could be largely divided into sporulation (*codY*, *sigE*, *sigK*, *soj*, *spo0A*, *spollAA*, *spollE*, CPF_2417, *ftsK*, *minD*, and *spoVD*) and biofilm formation (*ctrAB*, *abrB*, *luxS*, *sigG*, CPF_0368, *argG*, *ribD*, *ribE*, *lexA*, and *sleC*) [7,8,9,11,12,13]. The locations of SNPs in candidate genes, the role of SNPs in candidate genes, and the functional characteristics of candidate genes were identified. Using the Python module seaborn, the number of SNPs in the annotated CDS regions, the nonsynonymous SNP number, and the candidate gene-associated SNP count were indicated in scatter plots. The scatter plots were generated using Python (version 3.7.4) and Seaborn (version 0.10.1).

### 4.7. Extraction of RNA and RT-PCR

*C. perfringens* TYJAM-D-66, CMM-C-80, SDE-B-202, and ATCC 13124 strains were incubated overnight in TGY. A 2% inoculum was transferred to fresh medium, followed by incubation in six-well polystyrene plates (3 mL in each well) for 48 h. The sessile cells were collected for RNA extraction by removing the supernatant, and were washed with PBS one time, after which the sessile cells were detached from the surface with PBS through repetitive pipetting. The planktonic cells for the control were prepared with the overnight incubation in TGY broth. The RNA of harvested samples was purified using the PureLink RNA Mini Kit (Invitrogen, Carlsbad, CA, USA), and DNA was removed with Ambion DNase I (Invitrogen), according to the manufacturer’s instructions. The integrity of purified RNA was verified in 23S and 16S rRNA with 1.5% agarose gel electrophoresis (Supplementary Figure S1), quantified using a Nanodrop spectrophotometer (NanoDrop One, Thermo Fisher Scientific, Madison, WI, USA), and stored in a −80 °C freezer. Reverse transcription of total RNA 1 µg was performed with the Transcriptor First Strand cDNA Synthesis Kit (Thermo Fisher). The cDNA was then used as a template for PCR with primers targeting genes associated with biofilm formation and sporulation (Supplementary Table S2). The primer designing software was Primer Premier 5.0 (Premier Biosoft International Company, Palo Alto, CA, USA). RT–PCR was performed with SYBR green PCR master mix (Bio-Rad), and the conditions were as follows—95 °C for 2 min; 45 °C for 1 h; 35 cycles of 95 °C for 30 s, 55 °C for 40 s, and 72 °C for 40 s; and a single final extension at 72 °C for 5 min. The housekeeping gene 16S rRNA served as an internal control. All RT–PCR assays for each strain was performed in triplicates. The fold change was indicated as the relative change of identified genes of the expression levels in sessile cells compared to the planktonic cells.

### 4.8. Statistical Analysis

Data were subjected to statistical analyses using SPSS 12.0 (SPSS Inc., Chicago, IL, USA). The significant differences in each sample were analyzed by Duncan’s multiple range test and Student’s *t*-test at *p* < 0.05.

## 5. Conclusions

This investigation provides background data for the evaluation and application of disinfectants for the treatment of *C. perfringens*. The results expound the changes of isolates with different conditions. Whole-genome sequence analysis and genomes transcriptional profiles would be useful for the study of the different characteristic in the isolates. These results would be helpful for understanding the mechanism of biofilm formation and sporulation in *C. perfringens* isolates.

## Figures and Tables

**Table 1:** Fold changes in biofilm formation and sporulation-related gene transcription in *C. perfringens* biofilms.

Gene	Functional Annotation	Fold Change				Total SNP Counts Compared with ATCC 13124 (Nonsynonymous Counts)
		TYJAM-D-66	CMM-C-80	SDE-B-202	ATCC 13124	
**Biofilm formation**						
*ctrAB*	hypothetical protein	3.04	−3.43	−5.64	−1.99	ND ^1^
*abrB*	transcriptional regulator, AbrB family	0.36	−4.42	−4.13	−1.06	ND
*luxS*	autoinducer-2 production protein	17.06	6.60	−3.50	−3.58	10(1)
*sigG*	RNA polymerase sigma-G factor	44.98	−1.85	−0.30	4.05	ND
CPF_0368	rubrerythrin family protein	0.36	−0.76	−1.32	2.15	8(3)
*argG*	argininosuccinate synthase	113.48	2.30	2.73	1.99	27(4)
*ribD*	5-amino-6-(5-phosphoribosylamino) uracil reductase	42.78	−2.34	−1.03	3.19	32(17)
*ribE*	riboflavin synthase, alpha subunit	1.55	−1.72	−4.89	5.95	21(12)
*lexA*	transcriptional repressor	1.51	−1.11	−3.85	−1.95	14(3)
*sleC*	spore cortex-lytic enzyme	−4.66	−2.77	−3.83	5.49	ND
**Sporulation**						
*codY*	GTP-sensing transcriptional pleiotropic repressor	9.47	−1.79	−1.95	−2.89	ND
*sigE*	RNA polymerase sigma-E factor	23.20	−0.35	−4.27	−3.00	10
*sigK*	RNA polymerase sigma-70 factor family	70.16	−1.67	−4.73	1.11	ND
*soj*	sporulation initiation inhibitor	3.21	1.01	−3.40	−1.22	5(1)
*spo0A*	sporulation master regulation	2.16	−3.02	−4.77	1.96	3
*spollAA*	sporulation master regulation	22.14	−2.09	−4.47	3.31	ND
*spollE*	anti-sigma F factor antagonist	58.15	−1.80	−5.07	9.36	ND
CPF_2417	small, acid-soluble spore protein	11.64	−2.19	−3.54	6.70	1
*ftsK*	DNA translocase	5.96	−2.40	−3.73	−1.04	36(7)
*minD*	septum site-determining protein	4.89	−2.33	−3.92	−1.99	5
*spoVD*	stage V sporulation protein D	23.02	2.49	−1.98	2.49	ND

^1^ ND: not detected.

**Table 2:** Toxin gene profiles of *C. perfringens* isolates [6].

Strains	Toxin Gene				Toxinotype
	*cpa*	*cpe*	*cpb*2	*netB*	
TYJAM-D-66	+	+	−	−	F
CMM-C-80	+	−	−	+	G
SDE-B-202	+	+	−	−	F
ATCC 13124	+	−	−	−	A

## Data Availability

The data presented in this study are available on request from the corresponding author.

## Supplementary Materials

The Supplementary table is available online at https://www.mdpi.com/article/10.3390/antibiotics10040396/s1. Table S1. The list of SNPs of the *C. perfringens* isolates and ATCC 13124. Table S2. Primers used in this study. Figure S1. Agarose gel electrophoresis of the total RNA samples.

## References

[1] Navarro M.A., McClane B.A., Uzal F.A. (2018). Mechanisms of action and cell death associated with *Clostridium perfringens* toxins. Toxins.

[2] Uzal F.A., Freedman J.C., Shrestha A., Theoret J.R., Garcia J., Awad M.M., Adams V., Moore R.J., Rood J.I., Mcclane B.A. (2014). Towards an understanding of the role of *Clostridium perfringens* toxins in human and animal disease. Future Microbiol..

[3] Uzal F.A., Navarro M.A., Li J., Freedman J.C., Shrestha A., McClane B.A. (2018). Comparative pathogenesis of enteric clostridial infections in humans and animals. Anaerobe.

[4] Shrestha A., Uzal F.A., McClane B.A. (2018). Enterotoxic clostridia: *Clostridium perfringens* enteric diseases. Microbiol. Spectr..

[5] Charlebois A., Jacques M., Boulianne M., Archambault M. (2017). Tolerance of *Clostridium perfringens* biofilms to disinfectants commonly used in the food industry. Food Microbiol..

[6] Hu W.S., Kim H., Koo O.K. (2018). Molecular genotyping, biofilm formation and antibiotic resistance of enterotoxigenic *Clostridium perfringens* isolated from meat supplied to school cafeterias in South Korea. Anaerobe.

[7] Majed R., Faille C., Kallassy M., Gohar M. (2016). *Bacillus cereus* biofilms-same, only different. Front. Microbiol..

[8] Li J., Ma M., Sarker M.R., McClane B.A. (2013). CodY is a global regulator of virulence-associated properties for *Clostridium perfringens* type D strain CN3718. mBio.

[9] Ohtani K., Hirakawa H., Paredes-Sabja D., Tashiro K., Kuhara S., Sarker M.R., Shimizu T. (2013). Unique regulatory mechanism of sporulation and enterotoxin production in *Clostridium perfringens*. J. Bacteriol..

[10] Varga J.J., Therit B., Melville S.B. (2008). Type IV pili and the CcpA protein are needed for maximal biofilm formation by the gram-positive anaerobic pathogen *Clostridium perfringens*. Infect. Immun..

[11] Li J., McClane B.A. (2010). Evaluating the involvement of alternative sigma factors SigF and SigG in *Clostridium perfringens* sporulation and enterotoxin synthesis. Infect. Immun..

[12] García T.G., Ventroux M., Derouiche A., Bidnenko V., Santos S.C., Henry C., Mijakovic I., Noirot-Gros M.F., Poncet S. (2018). Phosphorylation of the *Bacillus subtilis* replication controller YabA plays a role in regulation of sporulation and biofilm formation. Front. Microbiol..

[13] Charlebois A., Jacques M., Archambault M. (2016). Comparative transcriptomic analysis of *Clostridium perfringens* biofilms and planktonic cells. Avian Pathol..

[14] Sanchez-Salas J.L., Setlow B., Zhang P., Li Y., Setlow P. (2011). Maturation of released spores is necessary for acquisition of full spore heat resistance during *Bacillus subtilis* sporulation. Appl. Environ. Microbiol..

[15] Vidal J.E., Shak J.R., Canizalez-Roman A. (2015). The CpAL quorum sensing system regulates production of hemolysins CPA and PFO to build *Clostridium perfringens* biofilms. Insect Infect. Immun..

[16] Dapa T., Leuzzi R., Ng Y.K., Baban S.T., Adamo R., Kuehne S.A., Scarselli M., Minton N.P., Serruto D., Unnikrishnan M. (2013). Multiple factors modulate biofilm formation by the anaerobic pathogen *Clostridium difficile*. J. Bacteriol..

[17] Mi E., Li J., McClane B.A. (2018). NanR regulates sporulation and enterotoxin production by *Clostridium perfringens* type F strain F4969. Infect. Immun..

[18] Hamon M.A., Lazazzera B.A. (2001). The sporulation transcription factor Spo0A is required for biofilm development in *Bacillus subtilis*. Mol. Microbiol..

[19] Simões M., Simões L.C., Vieira M.J. (2010). A review of current and emergent biofilm control strategies. LWT Food Sci. Technol..

[20] Talukdar P.K., Udompijitkul P., Hossain A., Sarker M.R. (2017). Inactivation strategies for *Clostridium perfringens* spores and vegetative cells. Appl. Environ. Microb..

[21] Zhao Y., Melville S.B. (1998). Identification and characterization of sporulation-dependent promoters upstream of the enterotoxin gene (*cpe*) of *Clostridium perfringens*. J. Bacteriol..

[22] Castro M.G., Icazatti A., Divizia J., Vega A.E., Cortiñas T.I., Stagnitta P.V. (2016). Effect of sanitizers and glucose on *Clostridium perfringens* biofilm formation and growth. Int. J. Curr. Microbiol. Appl. Sci..

[23] Li J., Paredes-Sabja D., Sarker M.R., McClane B.A. (2016). *Clostridium perfringens* sporulation and sporulation-associated toxin production. Microbiol. Spectrum.

[24] Pantaleon V., Bouttier S., Soavelomandroso A.P., Janoir C., Candela T. (2014). Biofilms of *Clostridium* species. Anaerobe.

[25] Wiencek K.M., Klapes N.A., Foegeding P.M. (1990). Hydrophobicity of *Bacillus* and *Clostridium* spores. Appl. Environ. Microb..

[26] Semenyuk E.G., Laning M.L., Foley J., Johnston P.F., Knight K.L., Gerding D.N., Driks A. (2014). Spore formation and toxin production in *Clostridium difficile* biofilms. PLoS ONE.

[27] Briolat V., Reysset G. (2002). Identification of the *Clostridium perfringens* genes involved in the adaptive response to oxidative stress. J. Bacteriol..

[28] Jean D., Briolat V., Reysset G. (2004). Oxidative stress response in *Clostridium perfringens*. Microbiology.

[29] Chen J., Wang Y. (2020). Genetic determinants of *Salmonella enterica* critical for attachment and biofilm formation. Int. J. Food Microbiol..

[30] Charlebois A., Jacques M., Archambault M. (2014). Biofilm formation of *Clostridium perfringens* and its exposure to low-dose antimicrobials. Front. Microbiol..

[31] Beuchat L.R., Pettigrew C.A., Tremblay M.E., Roselle B.J., Scouten A.J. (2005). Lethality of chlorine, chlorine dioxide, and a commercial fruit and vegetable sanitizer to vegetative cells and spores of *Bacillus cereus* and spores of *Bacillus thuringiensis*. J. Ind. Microbiol. Biot..

[32] Behnke S., Parker A.E., Woodall D., Camper A.K. (2011). Comparing the chlorine disinfection of detached biofilm clusters with sessile biofilms and planktonic cells in single and dual species cultures. Appl. Environ. Microb..

[33] Edwards A.N., Karim S.T., Pascual R.A., Jowhar L.M., Anderson S.E., McBride S.M. (2016). Chemical and stress resistances of *Clostridium difficile* spores and vegetative cells. Front. Microbiol..

[34] Paredes-Sabja D., Sarker N., Setlow B., Setlow P., Sarker M.R. (2008). Roles of DacB and Spm proteins in *Clostridium perfringens* spore resistance to moist heat, chemicals, and UV radiation. Appl. Environ. Microb..

[35] Paredes-Sabja D., Setlow B., Setlow P., Sarker M.R. (2008). Characterization of *Clostridium perfringens* spores that lack SpoVA proteins and dipicolinic acid. J. Bacteriol..

[36] Elzain A.M., Elsanousi S.M., Ibrahim M.E.A. (2019). Effectiveness of ethanol and methanol alcohols on different isolates of *staphylococcus* species. J. Bacteriol. Mycol..

[37] Walter B.M., Cartman S.T., Minton N.P., Butala M., Rupnik M. (2015). The SOS response master regulator LexA is associated with sporulation, motility and biofilm formation in *Clostridium difficile*. PLoS ONE.

[38] Kampf J., Gerwig J., Kruse K., Cleverley R., Dormeyer M., Grünberger A., Kohlheyer D., Commichau F.M., Lewis R.J., Stülke J. (2018). Selective pressure for biofilm formation in *Bacillus subtilis*: Differential effect of mutations in the master regulator SinR on bistability. mBio.

[39] Yan F., Yu Y., Wang L., Luo Y., Guo J.H., Chai Y. (2016). The *comER* gene plays an important role in biofilm formation and sporulation in both *Bacillus subtilis* and *Bacillus cereus*. Front. Microbiol..

[40] Pagedar A., Singh J. (2012). Influence of physiological cell stages on biofilm formation by *Bacillus cereus* of dairy origin. Int. Dairy J..

[41] Burton B.M., Marquis K.A., Sullivan N.L., Rapoport T.A., Rudner D.Z. (2007). The ATPase SpoIIIE transports DNA across fused septal membranes during sporulation in *Bacillus subtilis*. Cell.

[42] Jones S.W., Paredes C.J., Tracy B., Cheng N., Sillers R., Senger R.S., Papoutsakis E.T. (2008). The transcriptional program underlying the physiology of clostridial sporulation. Genome Biol..

[43] Hardie K.R., Heurlier K. (2008). Establishing bacterial communities by ‘word of mouth’: LuxS and autoinducer 2 in biofilm development. Nat. Rev. Microbiol..

[44] Zhao X., Liu R., Tang H., Osei-Adjei G., Xu S., Zhang Y., Huang X. (2018). A 3’ UTR-derived non-coding RNA RibS increases expression of *cfa* and promotes biofilm formation of *Salmonella enterica* serovar Typhi. Res. Microbiol..

[45] Stasiewicz M.J., Oliver H.F., Wiedmann M., den Bakker H.C. (2015). Whole-genome sequencing allows for improved identification of persistent *Listeria monocytogenes* in food-associated environments. Appl. Environ. Microb..

[46] Quainoo S., Coolen J.P.M., van Hijum S.A.F.T., Huynen M.A., Melchers W.J.G., van Schaik W., Wertheim H.F.L. (2017). Whole-genome sequencing of bacterial pathogens: The future of nosocomial outbreak analysis. Clin. Microbiol. Rev..

[47] Mac Aogáin M., Moloney G., Kilkenny S., Kelleher M., Kelleghan M., Boyle B., Rogers T.R. (2015). Whole-genome sequencing improves discrimination of relapse from reinfection and identifies transmission events among patients with recurrent Clostridium difficile infections. J. Hosp. Infect..

[48] Ronco T., Stegger M., Ng K.L., Lilje B., Lyhs U., Andersen P.S., Pedersen K. (2017). Genome analysis of *Clostridium perfringens* isolates from healthy and necrotic enteritis infected chickens and turkeys. BMC Res. Notes.

[49] Sokurenko E.V., Hasty D.L., Dykhuizen D.E. (1999). Pathoadaptive mutations: Gene loss and variation in bacterial pathogens. Trends Microbiol..

[50] Myers G.S.A., Rasko D.A., Cheung J.K., Ravel J., Seshadri R., DeBoy R.T., Ren Q., Varga J., Awad M.M., Brinkac L.M. (2006). Skewed genomic variability in strains of the toxigenic bacterial pathogen, *Clostridium perfringens*. Genome Res..

