# Supplementary material for: Biofilm and Spore Formation of Clostridium perfringens and Its Resistance to Disinfectant and Oxidative Stress
Source: Antibiotics (Basel). 2021 Apr 6;10(4):396. doi: 10.3390/antibiotics10040396 (PMC8067515; doi:10.3390/antibiotics10040396)
Supplement: Supplementary file 1 [file antibiotics-10-00396-s001.zip › Supplementary_Table S1_210113.docx]

Supplement

Table S1. RT-PCR primers used in this experiment.

| Gene | Forward / Reverse | Primer Sequence (5’→3’) | Product length (bp) | Reference |
| --- | --- | --- | --- | --- |
| 16S RNA | 16sF | CCTTACCTACACTTGACATCCC | 119 | [8] |
|  | 16sR | GGACTTAACCCAACATCTCACG |  |  |
| *ctrAB* | F | ACTGGGGAGAAAATAAAAAGAGCTA | 143 | This study |
|  | R | GCAACTAACTCTAGCACCCA |  |  |
| *abrB* | F | CAGGTGTAGTAAGAAGAGTAGACGA | 98 | This study |
|  | R | GATTTCTAAAGCATCCTTTTCTGCT |  |  |
| *luxS* | F | TCACCAATGGGATGCAAAACAG | 89 | This study |
|  | R | GCTGCTGGTATTTCTTCTTGCT |  |  |
| *sigG* | F | TGATGTTCTTCCAGCACCTCTT | 82 | This study |
|  | R | TCTAATAGCTTCATCATTCTCCCC |  |  |
| CPF_0368 | F | CAAGGAGAATTAGATGCAGTTCTTG | 101 | This study |
|  | R | TTCCTTCATCTGCTGCTATGCT |  |  |
| *argG* | F | CTACCATACAAGCTGGGGCAA | 127 | This study |
|  | R | CCATGACAAATAGCATCTGCACC |  |  |
| *ribD* | F | AGAGAGTCAGGCATAGAGGTT | 125 | This study |
|  | R | TCCATCTAAGGTCATTGCCCA |  |  |
| *ribE* | F | GAGGGGGAAGCAATATGGGTAG | 122 | This study |
|  | R | CTTTCCCCTTTAACCTCTGCAA |  |  |
| *lexA* | F | ACTGCTGGTATGCCTATTTTAGC | 128 | This study |
|  | R | CCGGCTTCTATCATACTGTCTCC |  |  |
| *sleC* | F | CCTAAGGTTGCTGTAGACGGG | 100 | This study |
|  | R | AATCCACCTCTCCTGTTTGAGG |  |  |
| *codY* | F | TGATGATGACCTAGTTTTAGCAGA | 118 | This study |
|  | R | AGCTAATTGAACCACCGCTT |  |  |
| *sigE* | F | AAGCTTGCAACCTATGCATCAA | 102 | This study |
|  | R | TCCATCCCAGTCTATATTTAAGGGC |  |  |
| *sigK* | F | ACCAAAACTCAAAAGAAATGGATGA | 112 | This study |
|  | R | GCTGCATATGTTGCAAGTCGT |  |  |
| *soj* | F | GCTCCGAGTTTTGGATTGCC | 104 | This study |
|  | R | TCACTCCCTGTCTTTTTAGGAA |  |  |
| *spo0A* | F | TTGCTTGGGGAAGAGGACAAA | 88 | This study |
|  | R | TCTGAGTTTGTTGGTTTACCCTTA |  |  |
| *spollAA* | F | TCATAGTGCAGAAGAAGTAAGAGT | 117 | This study |
|  | R | ACACCAATTCCTGAACTATCCA |  |  |
| *spollE* | F | AGCAGTTGCTACTAAAGAGGGA | 113 | This study |
|  | R | CAGGTCCTGATCCCATGCC |  |  |
| CPF_2417 | F | AACGAGGTAGCTAGAGAATTAGGTG | 98 | This study |
|  | R | CTTTTTACCATTTCGCCGCCT |  |  |
| *ftsK* | F | TCAGAGGGAAATGGTGATGGTG | 84 | This study |
|  | R | TGCTGAGGCTTGACCACTTT |  |  |
| *minD* | F | ACCTACTGCTCAAACAAAGGAT | 112 | This study |
|  | R | ACCAGCTGGGCAATCTAAAA |  |  |
| *spoVD* | F | TGTTTCAGACTCCTTCGAACCT | 80 | This study |
|  | R | TACCAGCTACCCCTTCTTGGA |  |  |
