# Supplementary figures and images for: Biofilm and Spore Formation of Clostridium perfringens and Its Resistance to Disinfectant and Oxidative Stress
Source: Antibiotics (Basel). 2021 Apr 6;10(4):396. doi: 10.3390/antibiotics10040396 (PMC8067515; doi:10.3390/antibiotics10040396)

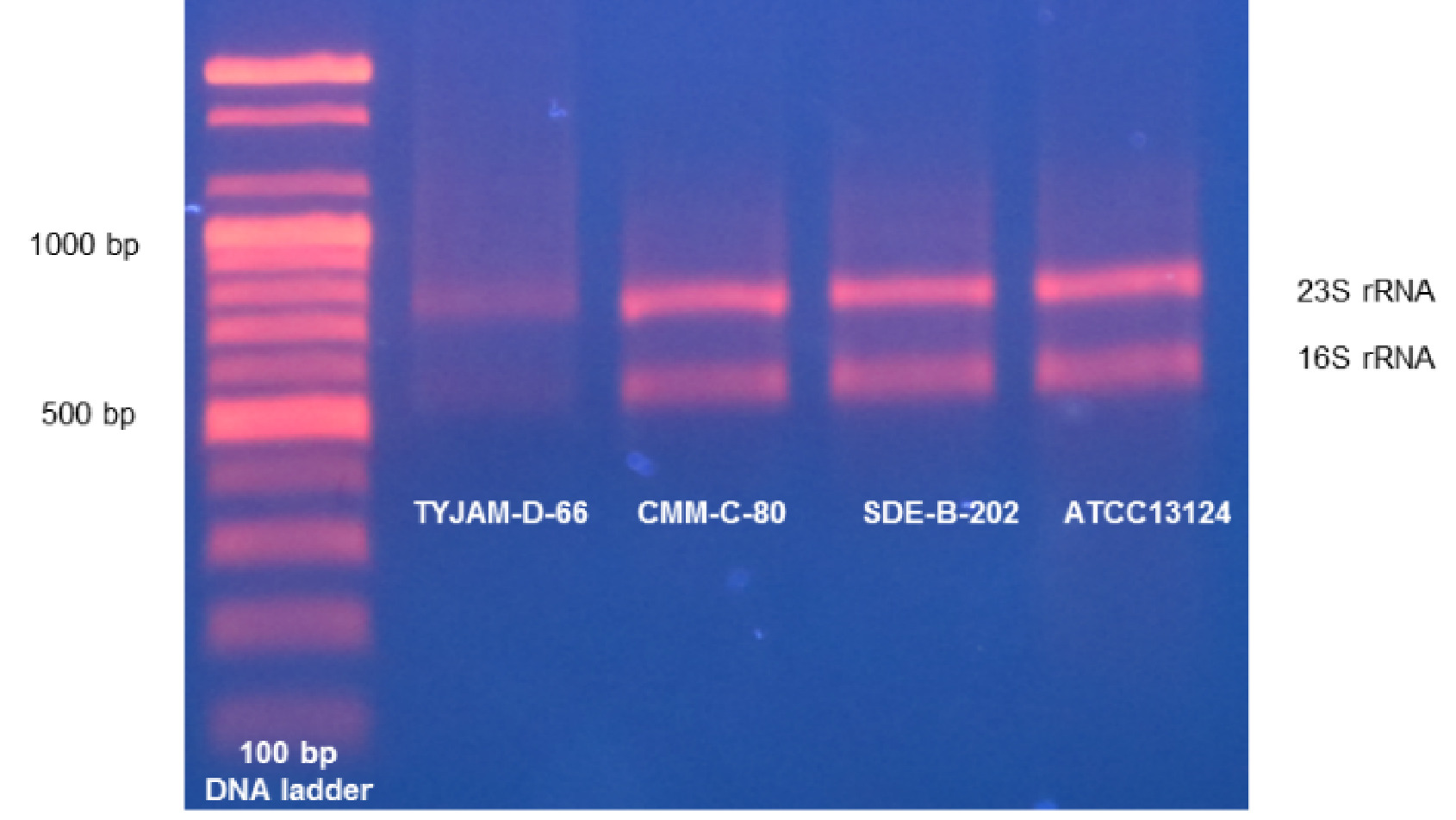

Supplement: Supplementary file 1 [file antibiotics-10-00396-s001.zip › Figure_S1_210125.jpg]
